# Synergistic Effect of Pressurization Rate and β-Form Nucleating Agent on the Multi-Phase Crystallization of iPP

**DOI:** 10.3390/polym13172984

**Published:** 2021-09-03

**Authors:** Wenxia Jia, Ranran Zhuo, Mingkun Xu, Jiaxiang Lin, Xiaoting Li, Chuntai Liu, Changyu Shen, Chunguang Shao

**Affiliations:** 1 Key Laboratory of Materials Processing and Mold, National Engineering Research Center for Advanced Polymer Processing Technology, Ministry of Education, Zhengzhou University, Zhengzhou 450002, China; zzdx2019628@163.com (W.J.); xumkofmail@163.com (M.X.); LJX950727@126.com (J.L.); zdlixt@163.com (X.L.); ctliu@zzu.edu.cn (C.L.); shency@zzu.edu.cn (C.S.); 2 School of Physics and Microelectronics, Zhengzhou University, Zhengzhou 450002, China; zhuoran242@zzu.edu.cn

**Keywords:** isotactic polypropylene, pressurization rate, β-form nucleating agent, multi-phase crystallization

## Abstract

Using a homemade pressure device, we explored the synergistic effect of pressurization rate and β-form nucleating agent (β-NA) on the crystallization of an isotactic polypropylene (iPP) melt. The obtained samples were characterized by combining small angle X-ray scattering and synchrotron wide angle X-ray diffraction. It was found that the synergistic application of pressurization and β-NA enables the preparation of a unique multi-phase crystallization of iPP, including β-, γ- and/or mesomorphic phases. Pressurization rate plays a crucial role on the formation of different crystal phases. As the pressurization rate increases in a narrow range between 0.6–1.9 MPa/s, a significant competitive formation between β- and γ-iPP was detected, and their relative crystallinity are likely to be determined by the growth of the crystal. When the pressurization rate increases further, both β- and γ-iPP contents gradually decrease, and the mesophase begins to emerge once it exceeds 15.0 MPa/s, then mesomorphic, β- and γ- iPP coexist with each other. Moreover, with different β-NA contents, the best pressurization rate for β-iPP growth is the same as 1.9 MPa/s, while more β-NA just promotes the content of β-iPP under the rates lower than 1.9 MPa/s. In addition to inducing the formation of β-iPP, it shows that β-NA can also significantly promote the formation of γ-iPP in a wide pressurization rate range between 3.8 to 75 MPa/s. These results were elucidated by combining classical nucleation theory and the growth theory of different crystalline phases, and a theoretical model of the pressurization-induced crystallization is established, providing insight into understanding the multi-phase structure development of iPP.

## 1. Introduction

Isotactic polypropylene (iPP) possesses a specific position among semicrystalline polymers due to its comprehensive physical and chemical properties and good processing performance [1,2]. Its rich polymorphism, which has received substantial attention in scientific research and industrial applications, has a high effect on the properties [3,4]. iPP can form several crystal modifications including monoclinic α-iPP, trigonal β-iPP, orthorhombic γ-iPP, and mesophase (intermediate state between ordered and amorphous phase), all sharing the same threefold conformation, but with different spatial arrangements of chains in the crystal lattice [5,6,7,8,9,10,11]. Though the most common crystalline phase of iPP is α phase when its melt solidifies under ordinary conditions, due to the presence of the lamellae interlocking effect, α-iPP usually shows inferior ductility, which greatly limits its application. On the contrary, due to the absence of the special cross-hatching lamellar structure, β-iPP allows the initiation and propagation of plastic deformation more easily and then enhances the energy dissipation during deformation, so that it shows excellent toughness (including enhanced impact strength and improved elongation at break). Thus, the polymorphic crystallization of iPP has received substantial attention [12,13,14,15].

The addition of nucleating agents (NAs) is the most effective method to develop polymorphic crystallization of iPP [15,16,17,18]. Usually, NAs can decrease the nucleation activation energy during the crystallization process, leading to the crystallization occurring at relatively higher temperature (lower supercooling). Different kinds of NAs could promote the formation of the corresponding crystal phase according to the epitaxy theory, but this seems different in some circumstances, such as the crystallization under high pressure. Sowinski et al. have shown that under high pressure, iPP can be efficiently nucleated in the γ-form by poly(tetrafluoroethylene) and Hyperform HPN-20E, both of which are known to nucleate the strong crystallization of iPP in the α-form at atmospheric pressure [19]. Zapala et al. demonstrated that under high pressure, the γ-iPP could be promoted by active heterogeneities, which were able to nucleate in α-form [20]. In addition, we also found that multi-walled carbon nanotubes, a typical α-NA, could promote the formation of γ-iPP under high pressure treatment, and successfully reinforce the products’ modulus and yield strength [21]. A reasonable explanation for these phenomena was pointed by Sowinski et al. [22], who said the high pressure facilitated the γ/α epitaxy growth in iPP, where the primary nucleation occurred in the α-form on the surface of α-NAs, followed by the growth of the γ crystal. These results indicate that there was competition between the effect of α-NAs, which promoted the formation of α phase, and the effect of high pressure that promoted the growth of γ-iPP.

It is well known that pressure could lead to an increase in equilibrium melting temperature of iPP and thus obtain the extra undercooling, which means that pressure may vary the nucleation density and the growth rate of different crystals [9,23,24,25]. Moreover, pressure is favorable for the growth of γ-iPP since the γ phase has lower free energy than other crystal modifications at the elevated pressure [26,27]. Phillips and coworkers reported that the γ-iPP starts to form at a low pressure of about 50 MPa and becomes dominant at a high pressure of about 200 MPa, accompanied by the decrease in α-iPP [9]. Yang et al. have showed that pressure and flow can jointly induce “oriented” iPP spherulites consisting of α- and γ-modifications, while a high pressure was beneficial for γ-iPP formation, but flow brings an opposite effect [14]. Recently, we have observed the competitive formation of γ-iPP and mesophase under high-pressure treatment and presented the special dependence on the pressure and pressurization rate [28]. It has also been proven that the pressurization method can get rid of thermal conductivity of the melt and would produce products with a more uniform structure with a larger size, which seems effective to optimize the mechanical properties of polymer products [29,30].

When NAs and pressure treatment take effect simultaneously, an effective approach may be developed to improve iPP polymorphic crystallization [12,19,31]. However, as mentioned above, till now most of the research focuses on iPP mixed with α-NAs, but few reports have devoted to the effects of pressure on crystallization of iPP containing β-NAs. Obadal et al. proposed that aryl amide derivative (a kind of β-NA) enhanced the formation of the γ-iPP during isothermal crystallization at a lower pressure or at a high pressure but low temperature [31]. Yang et al. reported an increase in γ-iPP and a decrease in β-iPP with increasing pressure in iPP nucleated with aryl amide derivative, and they found γ-iPP could grow on the lateral of the β-NA needlelike structure and attributed it to the dual nucleation effects of β-NA [32]. However, Sowinski et al. reported that calcium pimelate, another typical kind of β-NA, only has a slight effect on the crystallization of γ-iPP under a high pressure [33]. Despite these contributions, the debates are still apparent, and the crucial question is whether β-NA can promote the formation of γ-iPP and whether there exists a competition between β- and γ-iPP under high pressure treatment [19]. To our knowledge, the crystallization behavior of iPP under the synergistic effect of β-NA and pressurization treatment has not been investigated before.

In the current work, we studied the crystalline structure of iPP mixed with β-NA (rare earth nucleator) crystallized under a widely-ranging pressurization rate from 0.6–750 MPa/s. For the first time, we observed the competitive formation of β-, γ- and mesomorphic iPP during the pressurization treatment, and displayed their relative content changes as a function of pressurization rate. Moreover, we revealed the versatile role of β-NA in the formation of β- and γ-iPP by combining nucleation and growth theory of different crystalline phases. Furthermore, a theoretical model was given to illustrate the solidification of iPP melts under pressurization treatment, providing insights into understanding the multiphase structure development of iPP induced by the synergistic effect of pressurization rate and β-NA.

## 2. Materials and Methods

### 2.1. Materials

The iPP material (grade T30s) used in this study has a weight average molecular weight Mw = 399 kg/mol and a polydispersity Mw/Mn = 4.6, which was kindly supplied by Dushanzi Petroleum Chemical Co, Korla, China. The rare earth nucleator (WBG-II), a typical kind of β-NA, was supplied by Guangdong Winner Functional Materials Co., Ltd. (Guangdong, China). β-NA is a heteronuclear dimetal complex of lanthanum and calcium with some specific ligands. Its general formula is Ca_x_La_1-x_(LIG1)_m_(LIG2)_n_, where x and 1-x are the proportions of Ca^2+^ and La^3+^ ions in the complex, while LIG1 and LIG2 are, respectively, a dicarboxylic acid and amidetype ligand with a coordination number of m and n.

### 2.2. Preparation of Samples

The iPP granules and β-NA were firstly dried in a vacuum oven at 80 °C for 8 h. To achieve a good dispersion of β-NA in iPP a two-step process was employed. Namely, a master batch of 5 wt% β-NA in iPP was first prepared through melt blending of β-NA and iPP in a Haake MiniLab II twin-screw extruder for 7 min at 30 rpm and 200 °C, and then the master batch was diluted with iPP to obtain the desired iPP/WBG-II composition (iPP with 0.2 and 0.5 wt% β-NA, marked as iPP0.2 and iPP0.5). After being pelletized, the composites were compression-molded into circular samples with a diameter of 24 mm and a thickness of 1 mm. For comparison, the circular samples without β-NA blending (marked as iPP0) were also prepared.

**Figure 1:**
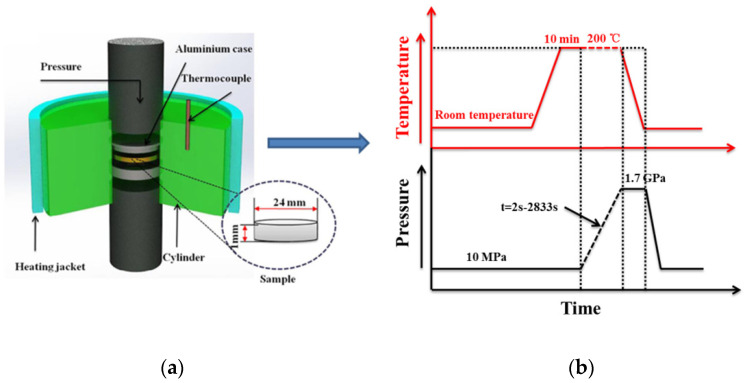
(**a**) Schematic of the high-pressure cell; (**b**) the temperature and pressure protocol.

The high-pressure treatment was performed on a homemade pressure device, and the sample assembly is shown in Figure 1a, whose detailed information has been described in previous work [28,34]. The experimental pressure and temperature profiles are shown in Figure 1b. The specimen was placed between two aluminum plates and pressed by two opposing pistons under a desired pressure within a certain time, and the required temperature was supplied by the outer heating jacket. The experimental procedure was as follows: after loading the specimen, a relatively low pressure (10 MPa) was applied, and the sample was heated to 200 °C and annealed at this temperature for 10 min to erase processing histories; in order to ensure that the sample has solidified at the end of pressurization, the desired pressure was set at 1.7 GPa so as to obtain a sufficiently high undercooling [28,34], then the relaxed melt at 200 °C were pressurized to 1.7 GPa within the controllable durations of 2, 22, 34, 45, 113, 226, 453, 895, 1308, 2125, and 2833 s, respectively, which correspond to the wide pressurization rate ranging from 0.6–750 MPa/s; subsequently, the pressurized samples were immediately cooled down to 40 °C at an average cooling rate of about 10 °C/min under 1.7 GPa; at last, the pressure was completely released to obtain the solidified samples. For comparison, another group of samples were prepared by heated to 200 °C, annealed for 10 min, and then cooled down to 40 °C under atmospheric pressure.

### 2.3. Characterizations

#### 2.3.1. Synchrotron Wide-Angle X-ray Diffraction

Synchrotron wide-angle X-ray diffraction (SR WAXD) measurements were carried out at the beamline BL16B of the Shanghai Synchrotron Radiation Facility (SSRF) with a wavelength (λ) of 0.124 nm. A Pilatus 200 K detector (487 × 407 pixels with a pixel size of 172 μm) was employed to collect the two-dimensional (2D) scattering patterns. The sample to detector distance was 227.3 mm. The exposure time was 10 s for each pattern. Fit2D software was used to analyze the data quantitatively. The 2D WAXD patterns were integrated to obtain the scattering intensity as a function of 2θ after correcting for background scattering from the air and sample holder.

The one-dimensional (1D) WAXD curves were fitted according to Gaussian functions to obtain the crystallinities of the samples. The phase content can be estimated by the following equation based on an interactive peak fit procedure [34]:

(1) $$X_{i}=A_{i}/\left(A_{\gamma }+A_{\beta }+A_{meso}+A_{amorp}\right)$$

where i represents the specific crystal phase (either γ, β, mesophase or amorphous) and $A_{i}$ represents the area of the fitted Lorentzian curve of the specific reflection. $A_{meso}$, $A_{\gamma }$, $A_{\beta },$ and $A_{amorp}$ are the fitted areas of mesomorphic, γ-, β- and amorphous phases, respectively. The crystalline size ($D_{hkl}$) determined in the direction perpendicular to (hkl) plane was estimated by using the Debye–Scherrer’s equation [3]:

(2) $$D_{hkl}=k\lambda /\beta_{hkl}cos\theta_{hkl}$$

where λ is the wavelength of the X-ray, k  is crystallite shape factor (0.89), $\theta_{hkl}$ is the Bragg angle, and $\beta_{hkl}$ is the full width at half maximum (FWHM) of the diffraction peak.

#### 2.3.2. Small-Angle X-ray Scattering

Small-angle X-ray scattering (SAXS) measurements were conducted using a Bruker Nanostar system. The operating voltage and current are 45 kV and 0.65 mA, respectively. SAXS patterns were recorded by a 2D vant 2000 detector. The wavelength of the X-ray used was 0.154 nm and the sample to detector distance was 1085 mm. The exposure time was 600 s for each scattering pattern.

Fit2D software was used to integrate the 2D SAXS results to 1D intensity distribution as a function of the module of scattering vector q=4πsinθ/λ with  2θ  being the scatter angle and  λ the X-ray wavelength. Through the 1D integrated curves, the peak position ($q_{max}$) can be obtained. According to the Bragg’s equation, the long period (L) of lamellae is calculated as follows:

(3) $$L=2\pi /q_{max}$$

## 3. Results

### 3.1. Crystallization of iPP and iPP/WBG-II under Atmospheric Pressure

**Figure 2:**
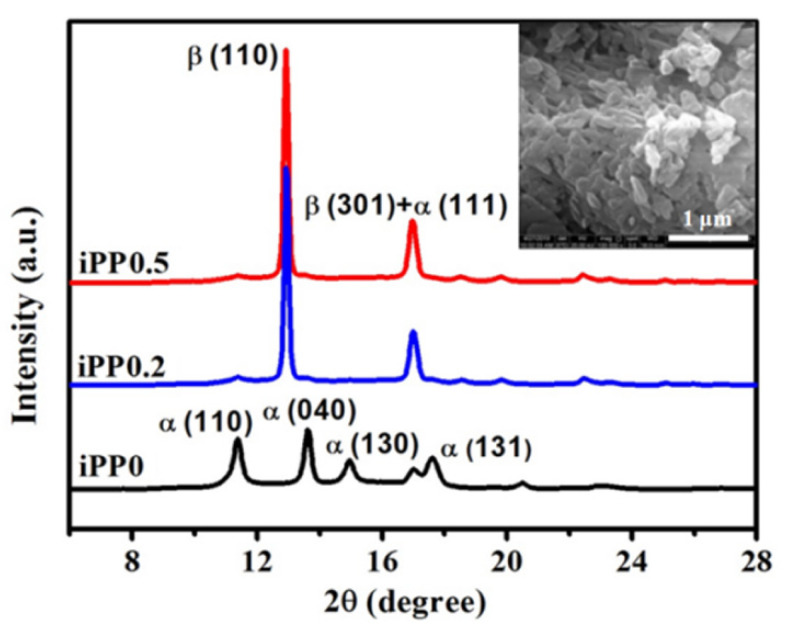
1D WAXD profiles of iPP0, iPP0.2, and iPP0.5 samples crystallized under atmospheric pressure (the inset shows scanning electronic microscopy micrograph of WBG-II, supplied by the provider).

Before high-pressure treatment, the crystalline structure and melting behavior of iPP0, iPP0.2, and iPP0.5 samples prepared by cooling under atmospheric pressure were characterized by SR WAXD, as shown in Figure 2. For iPP0 samples, the characteristic diffraction peaks of (110), (040), (130), (111), and (131) crystal planes of α-iPP were identified. On the other hand, iPP/WBG-II samples exhibit the typical characteristic diffraction peaks at 2θ = 12.9° and 17.0°, corresponding to (110) and (301) planes of β-iPP, and one weak diffraction peak of the α-iPP (110) plane can also be observed in iPP/WBG-II WAXD curves, which means a little amount of α-iPP was formed. This means that although WBG-II is an effective β-NA for iPP [35], it does not prevent the nucleation and growth of α-crystal. Moreover, there is no obvious difference between iPP0.2 and iPP0.5 WAXD curves, and the X_β_ of iPP0.2 and iPP0.5 are calculated to be 0.89 and 0.90, respectively.

### 3.2. Effect of Pressurization Rate on Polymorphic Crystallization of iPP0.2

**Figure 3:**
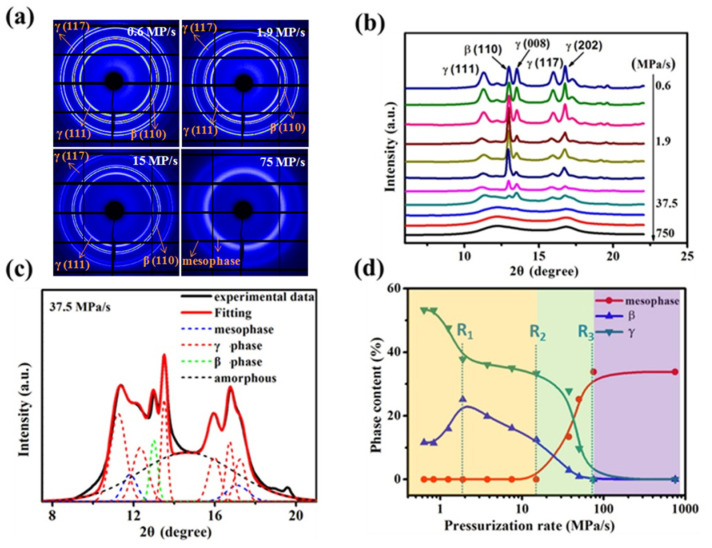
(**a**) Selected 2D WAXD patterns and (**b**) 1D WAXD profiles of iPP0.2 samples solidified by pressuring to 1.7 GPa at different pressurization rates; (**c**) the fitting curves of WAXD of amorphous, mesomorphic, β- and γ-phases; (**d**) fractions of different phases as a function of pressurization rates.

SR WAXD was used to detect the polymorphic composition of the high-pressure treated iPP0.2 samples. Figure 3a shows their 2D WAXD patterns, which shows that no orientation of the crystalline or amorphous phase was detected for all the samples, meaning the mold utilized in this experiment was well sealed and no flow occurred during the pressurization process [21]. Their 1D WAXD curves under different pressurization rates was given in Figure 3b, where no reflections corresponding to α-iPP were observed. At the lowest pressurization rate of 0.6 MPa/s, the characteristic diffraction peaks at 2θ = 11.3° (111), 13.5° (008), 16.0° (117), and 16.8° (202) attributed to γ-iPP can be observed, while the typical characteristic diffractions at 2θ = 12.9° for the β-iPP (110) plane was also identified. With the increasing of the pressurization rate, the intensity of (111), (008), (117), and (202) peaks decrease observably, demonstrating the content of the γ-iPP decrease remarkably. Differently, the intensity of β-iPP (110) increases at the initial pressurization rate increase and achieves the maximum as it reaches 1.9 MPa/s, then gradually reduces. Moreover, when the pressurization rate adds up higher than 75 MPa/s, all these crystal diffractions disappear and pure mesophase with two broad scattering halos at 2θ  of 11.9° and 17.0° appear.

To quantify the changes in crystallization polymorphism, the 1D WAXD signals were decomposed into specific contributions from γ-, β-, mesomorphic, and amorphous phases, see the representative fitting in Figure 3c [36], which displays a representative fitting performed on the WAXD profiles. Figure 3d presents the changes of the phase contents as a function of pressurization rate, and three critical rates (indicated by dotted lines) were identified. For simplified analysis, the amorphous phase is not taken into consideration. It can be seen that, at the lowest pressurization rate of 0.6 MPa/s, only β- and γ-iPP were obtained with the fractions of 11% and 54%, respectively. When the pressurization rate increases to 1.9 MPa/s (denoted as **R_1_**), it causes an obvious decrease in γ-iPP crystallinity from 54% to 40%, but the crystallinity of β-iPP increases visibly from 11% to the maximum point of about 24%. The increase in β-iPP crystallinity is almost equal to the decrease in γ-iPP crystallinity, meaning that the content of β-iPP increases at cost of the γ-iPP [25,37]. Once that rate exceeds **R_1_**, β-iPP and γ-iPP crystallinities both decrease gradually. When that rate further exceeds 15 MPa/s (denoted as **R_2_**), the mesophase begins to emerge, and mesomorphic, β-, and γ-iPP coexist with each other. After reaching 75 MPa/s (denoted as **R_3_**), pure mesophase could be obtained and its fraction content reaches the maximum of about 32%. It is worth noting that with pressurization, the formation of metastable structures has been observed for different kinds of polymers, and for iPP pressurization can induce more effects than temperature cooling on the formation of mesophase [38,39].

**Figure 4:**
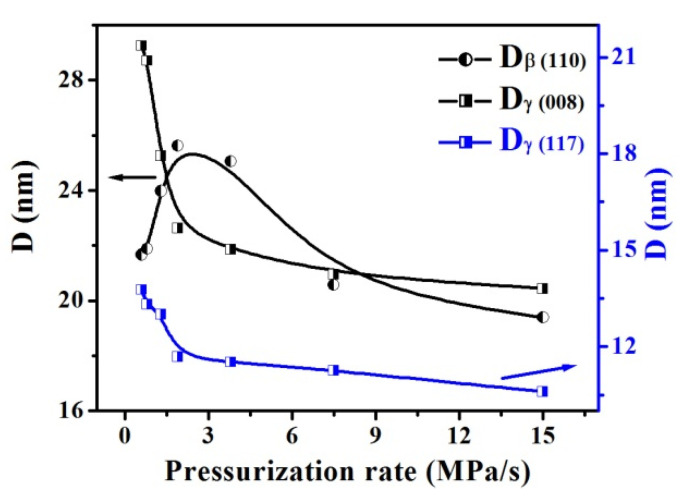
Changes in the crystallite size D for β and γ crystal as a function of pressurization rates.

Crystal perfection was studied to understand the formation of the β- and γ-phase induced by pressurization. Figure 4 displays the corresponding crystallite size D calculated by using the Debye–Scherrer’s equation. Clearly, within the range of 0.6 to 1.9 MPa/s (**R_1_**), D (008) of the γ crystal decreases rapidly from about 29.2 to 22.0 nm, and D (117) decreases from about 14.0 to 11.5 nm, indicating that the pressurization rate increasing greatly suppresses the growth of γ crystal. Instead, D (110) of the β crystal increases clearly from about 21.6 to 25.6 nm. With that rate enhanced greater than **R_1_**, both the γ and β crystals were restrained and their size reduces gradually. Interestingly, it can be found that, for the β crystal or γ crystal, the changing trend of its crystallite size is similar to that of its crystallinity (in Figure 3d); thus, we deduced that the crystallite size change is probable to induce the phase content change as well as the competitive formation between the β and γ phase.

**Figure 5:**
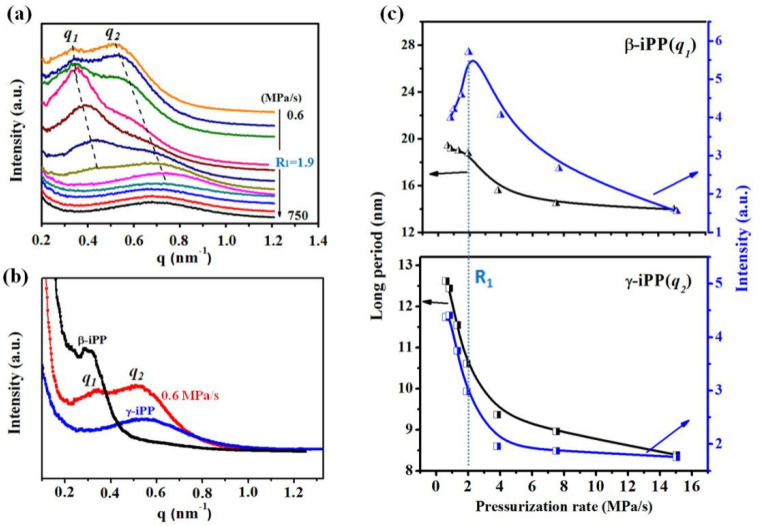
(**a**) Selected SAXS profiles of the iPP0.2 samples solidified by pressuring to 1.7 GPa at different pressurization rates; (**b**) Comparison of the SAXS profiles between pure β-, γ-iPP and the iPP0.2 samples prepared by pressuring at 0.6 MPa/s; (**c**) Changes in the maximal intensity of γ- and β-iPP as a function of pressurization rates.

SAXS was used to obtain the lamellae information. Figure 5a shows the Lorentz-corrected intensity plots as a function of scattering vector (*q* = 4π*sinθ*/λ). For the iPP0.2 sample treated at a rate of 0.6 MPa/s, two scattering peaks named $q_{1}$ and $q_{2}\;$ of 0.32 and 0.59 nm^−1^ were observed, which are close to that of pure β and γ crystal of iPP, respectively (Figure 5b). Figure 5c summarized the change trend of the maximum intensity ($I_{max}$) of the scattering peaks. With the increasing pressurization rate, the $I_{max}$ of the first peak adds in the early stage until it reaches **R_1_**, and then decreases observably, consistent with the change trend of β-iPP crystallinity (Figure 3d). Similarly, the $I_{max}$ of the second peak decreases with the pressurization rate increase, analogous to the change trend of γ-iPP content. Thereby, we attribute the first and second scattering peak to β- and γ-iPP lamellae stacks, respectively. Figure 5c also shows the evolution of relative long period L that is determined by using the Bragg law. The crystallized samples seem to have two divided long-period peaks, demonstrating that the crystals of β- and γ-iPP are present in the respective lamellae within different spherulites. It displays that when the pressurization rate increases from 0.6 MPa/s to **R_1_**, L of β crystal decreases slightly from about 19.0 to 18.0 nm, while the L of the γ crystal decreases remarkably from about 13.0 to 10.5 nm. The decrease in L corresponds to the decrease in lamellae thickness, meaning the small increase in the pressurization rate can suppress the growth of lamellae, and this inhibition effect in γ crystal seems more pronounced than that in β crystal. This behavior is consistent with the thermodynamic arguments made by Keller et al. and Troisi et al., which explained the lamellar thinning mechanism as being due to the pressure raised during crystallization [40,41]. We inferred that the crystallization would take place at a higher pressure under a higher pressurization rate, which would induce a higher supercooling degree for crystallization, thereby result in thinner lamellar thickness as well as the smaller crystallite size, as shown in Figure 4.

### 3.3. Synergistic Effect of Pressurization Rate and β-NA on Polymorphic Crystallization of iPP

**Figure 6:**
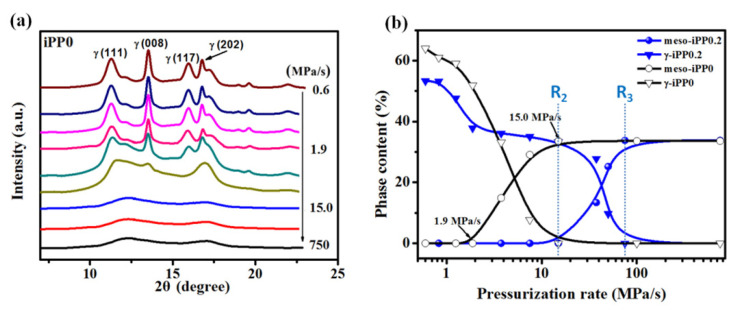
(**a**) Selected 1D WAXD profiles of iPP0 samples solidified by pressuring to 1.7 GPa at different pressurization rates; (**b**) Contents of β phase and γ phase as a function of pressurization rate.

In order to clarify the effect of β-NA on iPP crystallization behavior, the crystallization evolution of iPP0 and iPP0.5 melts under different pressurization rates were also studied, and their results were, respectively, compared with that of iPP0.2. Figure 6 displays the comparison of crystallization evolution in iPP0 and iPP0.2. For neat iPP, no β-iPP was induced by pressurization, and two threshold pressurization rates can be identified, of which the first one is about 1.9 MPa/s where mesophase starts to appear and the second one is about 15.0 MPa/s where only mesophase forms, see Figure 6a. In addition, the maximum γ-iPP crystallinity of about 63% can be obtained at the lowest pressurization rate of 0.6 MPa/s, indicating low pressurization rate was benefit for γ-iPP formation and a small increase in that rate can restrain that formation, leading to a significant reduction of its crystallinity. It should be noted that the corresponding threshold pressurization rates of the iPP0.2 (**R_2_** = 15 MPa/s, **R_3_** = 75 MPa/s) are much higher than that for neat iPP (1.9 MPa/s and 15 MPa/s, respectively) as shown in Figure 6b, and the shifts of the threshold values indicate that β-NA can promote the formation of γ-iPP crystal and thus suppress mesophase formation, especially in the pressurization rate range from 3.8 to 75 MPa/s.

**Figure 7:**
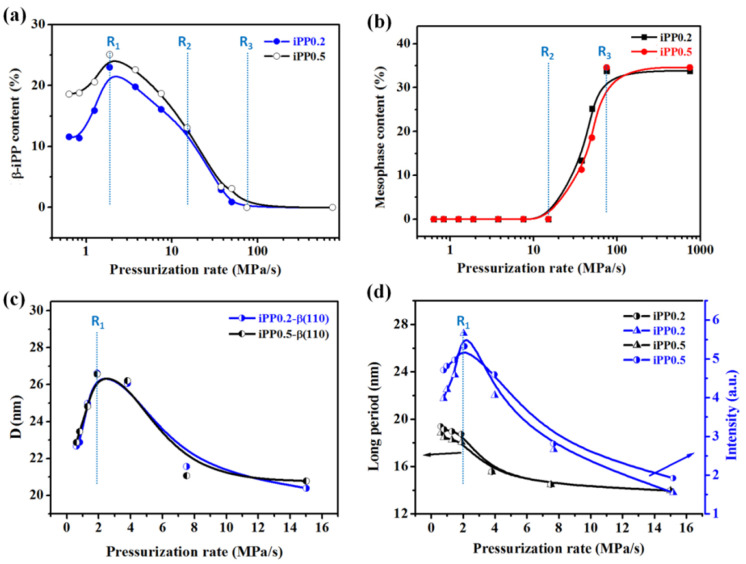
(**a**) Contents of β- and (**b**) mesomorphic phases of iPP0.2 and iPP0.5 as a function of pressurization rate; (**c**) Changes in D and (**d**) L, $I_{max}\;$ of β crystal in iPP0.2 and iPP0.5 samples with pressurization rates.

Figure 7 presents relative comparisons of iPP0.2 and iPP0.5 samples. Figure 7a shows that the change of different phase contents of iPP0.5 has a similar tendency as that of iPP0.2 with the pressurization rate changing, and three critical rates can also be identified. Further, the content of the β phase in iPP0.5 is obviously higher at the rate lower than **R_1_**, about 7% higher at 0.6 MPa/s for instance. However, the difference between their β-iPP contents gets smaller and even invisible after the rate reaches **R_2_**. These results indicated that more β-NA can provide more nucleated sites and promote the formation of β-iPP under the pressurization rate lower than **R_1_**, beyond which this promotion effect gradually diminishes. Additionally, the difference in the mesophase content is slight between iPP0.2 and iPP0.5, meaning β-NA content rarely has an effect on the formation of mesophase, see Figure 7b. Furthermore, the D (110) of iPP0.5 β crystals were also calculated as shown in Figure 7c, from which it can be seen that **R_1_** is also the dividing line, and its value coincides very well with that of iPP0.2. The long period and scattering intensity of iPP0.5 β crystals were displayed in Figure 7d, in which $I_{max}$ firstly increases and latterly decreases while L decreases monotonically, but slight differences were found with that of iPP0.2. As a result, a conclusion can be drawn that the growth of β-iPP crystal is solely controlled by the pressurization rate but is hardly influenced by β-NA content in the range used in this study.

## 4. Discussion

**Figure 8:**
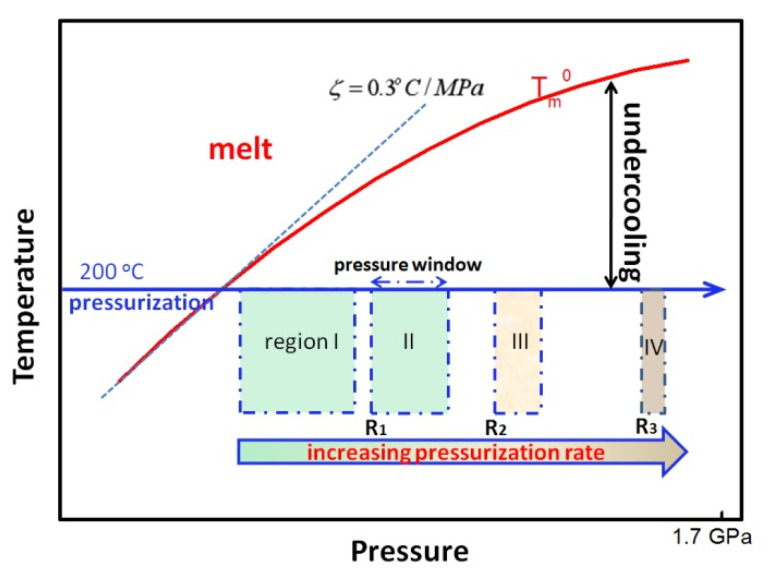
Theoretical model of the pressurization induced crystallization of β-NA blended iPP in the pressure–temperature diagram.

It is known that the thermodynamic effect of pressure elevation is an undercooling increase of a given crystalline form, which is the driving force for crystallization. Herein, for the polymers whose melting points increase with pressure, solidification condition of melts can be changed by adjusting pressurization rate, namely non-isobaric crystallization [42,43]. For iPP, the equilibrium melting temperature ($T_{m}^{0}$(*P*)) enhanced with the pressure increase, while the relation between iPP ($T_{m}^{0}$(*P*)) and pressure (*P*) usually can be expressed as follows [9]:

(4) $$T_{m}^{0}(P)=T_{m}^{0}+\zeta (P-P_{0})$$

where $P_{0}$ is the atmospheric pressure, $T_{m}^{0}$ is the equilibrium melting temperature at $P_{0}$. ζ is the pressure dependence of the melting temperature and could be applied as 0.3 °C/MPa at low pressure, while in this case, $T_{m}^{0}$(*P*) changes linearly with pressure. However, at a high pressure, ζ is not a constant but decreases with increasing pressure. Considering the experimental results of the literature, and in order to simplify the problem, we assume that β and γ phases share the same temperature and pressure dependence [25]. Therefore, the crystallization behavior of iPP melts under pressurization treatment can be correlated with the same equilibrium melting temperatures, and accordingly, the solidification of iPP melts could be divided into four regions labeled I, II, III, and IV, respectively, as shown in Figure 8.

A certain crystal fraction will only increase noticeably if the nuclei are appointed to this crystal phase and the growth rate of that phase is significant [25,44]. In region I, the pressurization rate is low, solidification of iPP melt will start at a low pressure. From the thermodynamics perspective, a low pressurization rate provides low undercooling, and the pressure window for the crystallization to take place is wide [10,14]. Therefore, the iPP melt tends to form γ-iPP which has better thermal stability and lower free energy than that of β-iPP, and the γ crystal will generate and grow into spherulites consisting of γ lamellae. Hence, the lowest pressurization rate corresponds to the maximum crystallinity of γ-iPP, agreeing well with Mezghani and Phillips’ report [45]. Meanwhile, from the kinetic perspective, the molecular chains have an enhanced ability of motion because of the low undercooling, hereafter benefitting from the growth of the γ crystal; thus, perfected γ crystals with thick lamellae will be obtained. However, the secondary nucleation can be simultaneously introduced by the addition of β-NA, which could provide a large quantity of nucleation sites for β crystal growth [18]. Hence, the competitive formation between β and γ crystal is present (Figure 3d).

In region II, the melts are treated by a higher pressurization rate, so that solidification should start at a higher pressure; then, a higher undercooling will be introduced, and the pressure window in which the crystallization occurs usually decreases. Two possible explanations are introduced to interpret the competitive crystallization behavior between β and γ crystals in this region. First, when the pressurization rate rises, it can significantly restrain and shorten the growth time of the γ crystal and lead to the sharp decrease in γ crystal size, which provides more space for β crystal growth. Second, it has been proven that there is an optimal undercooling for β crystal growth at atmosphere pressure in the temperature range of 100–135 °C where the β crystal can grow faster [44,46,47], while Van Erp and Peters et al. suggested that under high pressure a constant undercooling range of 60–90 °C is benefit for the growth of β crystal [48]. On this basis, we consider that the undercooling induced by the pressurization rate of **R_1_** is likely to be in the range of 60–90 °C favorable to β crystal growth [31]. In addition, as displayed in Figure 7c, the optical pressurization rate of **R_1_** for β crystal growth was not influenced by β-NA contents, which can further prove the correctness of this conjecture.

When a high pressurization rate (>**R_2_**) is used, a sufficient high undercooling and solidifying rate will be provided, and region III will be reached, where macromolecular chains in the melt has limited time to pack their segmental stems orderly. The nucleation behavior of the melt would change gradually from heterogeneous nucleation at a low supercooling to homogeneous nucleation at high supercooling [39,49,50], then metastable phase (or even amorphous) begins to be sought out and obeys the “law of successive states” [51,52]. However, with the existence of β-NA, the situation will be different from that of neat samples. NAs can induce the crystallization to take place at a lower pressure, namely at the lower supercooling, which is beneficial for the formation of γ-iPP. In this way, β-NA tends to suppress the generation of mesophase, and significantly promotes the formation of γ-iPP.

When the pressure increase is sufficiently high and rapid, as the pressurization rate rises greater than **R_3_**, region IV will be reached, where the pure mesophase can be obtained. Actually, for polymers whose melting point increases with increasing pressure, if the increase in pressure is high and rapid enough, the melt would be solidified to the metastable or even amorphous phase because of the sufficient undercooling induced by pressurization, and it coincides well with our other results [43,53,54].

## 5. Conclusions

In this work, the synergistic effect of pressurization rate (0.6–750 MPa/s) and β-NA (WBG-II) on the crystallization behavior of iPP were revealed by means of a homemade pressure device. The results show that the crystal formation of iPP strongly depends on the pressurization rate. The unique polymorphic iPP composed of β, γ, and/or mesomorphic phase can be prepared, and a function of these phase contents and the pressurization rate was established, where three critical pressurization rates marked as **R_1_**, **R_2_**, and **R_3_** were distinctly determined. Under the low pressurization rate range below **R_1_**, β- and γ-iPP grow competitively within different spherulites, while the best pressurization rate for β-iPP growth is **R_1_**. Once that rate exceeds **R_2_**, multiphase iPP including β, γ, and/or mesomorphic phases can be obtained, while pure mesophase can be obtained after it reaches **R_3_**. β-NA can significantly promote the formation of γ-iPP in a wide pressurization rate range between 3.8 to 75 MPa/s, and thereby suppress mesophase generation. The difference in β-NA content effect on crystallization is visible in low pressurization rate ranges lower than **R_1_**, where more nucleating agents promote more β-iPP. Finally, a theoretical model of the pressurization-induced crystallization was proposed, which offers a novel approach to develop polymorphic crystallization or further optimize mechanical properties of iPP products.

## Data Availability

Not applicable.
